# Characterization and function analysis of a novel gene, *Hc-maoc-1*, in the parasitic nematode *Haemonochus contortus*

**DOI:** 10.1186/s13071-017-1991-1

**Published:** 2017-02-06

**Authors:** Haojie Ding, Hengzhi Shi, Yu Shi, Xiaolu Guo, Xiuping Zheng, Xueqiu Chen, Qianjin Zhou, Yi Yang, Aifang Du

**Affiliations:** 1 College of Animal Sciences, Zhejiang Provincial Key Laboratory of Preventive Veterinary Medicine, Zhejiang University, Hangzhou, 310058 China; 2 Faculty of Life Science and Biotechnology, Ningbo University, Ningbo, 315211 China

**Keywords:** *Haemonchus contortus*, *Hc-maoc-1*, *Caenorhabditis elegans*, *Ce-maoc-1*, Diapause

## Abstract

**Background:**

Enoyl-CoA hydratase (MAOC) is required for the biosynthesis of the fatty acid-derive side chains of the ascaroside *via* peroxisome β-oxidation in the free-living nematode *Caenorhabditis elegans*. The derivative of dideoxy-sugar, ascarylose is used as dauer pheromones or daumones to induce development of the stress-resistant dauer larvae stage.

**Methods:**

*Hc-maoc-1* gene was obtained by searching the Wellcome Trusts Sanger Institute’s *H. contortus* genomic database. qRT-PCR was performed to analyse the transcriptional levels of *Hc-maoc-1* with different developmental stages as templates. IFA was carried out to determine the expression pattern in L3 larvae and micro-injection was used to verify the promoter activity of 5′-flanking region of *Hc-maoc-1*. Overexpression and RNAi experiments were applied in N_2_ strain to ascertain the gene function of *Hc-maoc-1*.

**Results:**

The full-length cDNA of *Hc-maoc-1* was 900 bp in length, which contained eight exons separated by seven introns and possessed the Hotdog domain and the MaoC-like domain, together with several other residues and a hydratase 2 motif. It was transcribed throughout the lifecycle and peaked in the fourth-stage larvae (L4) of *H. contortus*; however, its transcription level decreased in diapausing L4. The protein expression and location of Hc-MAOC-1 were mainly in the intestine of L3 larvae. Overexpression of *Ce-maoc-1* and *Hc-maoc-1* in *C. elegans* showed extended lifespan and increased body size. The protein Ce-MAOC-1 and Hc-MAOC-1 were localized in the intestine with a punctate pattern. In *C. elegans*, knockdown of *Ce-maoc-1* conferred shortened lifespan and body lengths, decreased brood size and increased lipid storage.

**Conclusion:**

*Caenorhabditis elegans* was used as a model organism to ascertain the function of *Hc-maoc-1* in *H. contortus*. Our results showed the similar characteristics and functions with *Ce-maoc-1* and provided evidences of the potential functions of *Hc-maoc-1* in biosynthesis of daumones in *H. contortus*.

**Electronic supplementary material:**

The online version of this article (doi:10.1186/s13071-017-1991-1) contains supplementary material, which is available to authorized users.

## Background

The gastric nematode *Haemonchus contortus* infects small ruminants (sheep and goats) worldwide, and causes great production losses. Infection by the infective third stage larvae (iL3) is seasonal. *Haemonchus contortus* may enter diapause to improve its population viability in harsh environment. Diapause is widespread in nematodes [1]. Diapause, a form of arrested development, occurs in the early fourth stage of *H. contortus* in abomasa of ruminants [2]. It is a strategy for this parasitic nematode to adapt to adverse environmental conditions such as low temperature in winter, low oxygen or immunoreaction of the infected host [1–3].

Dauer is a specialized stage in the free-living nematode *Caenorhabditis elegans*. It is regarded as a form of arrested L3 [4]. L2 stage of *C. elegans* would be induced to enter the arrest stage when larvae encounter hostile environments such as scarce food, high population density or high temperature [4–6]. The formation of dauer and recovery in *C. elegans* are precisely controlled by a constitutively produced ascaroside pheromones. Pheromones are regarded as the derivative of the dideoxy-sugar, ascarylose, which are consist of dideoxyhexose ascarylose and various short chain fatty acid moieties [7, 8]. Four peroxisomal enzymes participate in the ascaroside biosynthesis: acyl-CoA oxidase (ACOX-1), enoyl-CoA hydratase (MAOC-1), (3R)-hydroxyacyl-CoA dehydrogenase (DHS-28), and 3-ketoacyl-CoA thiolase (DAF-22) [9–12]. Nematodes carrying the gene *daf-22* mutants have been verified that are non-functioning in the biosynthesis of the dauer pheromone and male-attracting signals [13, 14]. ACOX-1 plays a role in ascaroside biosynthesis as a model: β-oxidation shortens long-chain ω/(ω-1)-ascarosides to short-chain ω/(ω-1)-ascarosides [15]. MAOC-1 and DHS-28 are homologues to human MFE-2 and control biosynthesis of different ascarosides in *C. elegans* [12]. Although the role of peroxisome in the dauer stage has been well studied, and great progress has been made in understanding its molecular mechanisms in *C. elegans*, the analogous process in the *H. contortus* iL3 is poorly understood. *Caenorhabditis elegans* and *H. contortus* evolutionarily belong to the clade V [16]. So, it is proposed that mechanisms used to determine entry into dauer in *C. elegans* and diapause in *H. contortus* are similar [17].

The purpose of the current study was to characterize Hc-MAOC-1 and to explode its function in *H. contortus*. In this study, we first characterized the complete cDNA of *Ce-maoc-1* orthologue in *H. contortus* and named as *Hc-maoc-1*. The localization of protein Hc-MAOC-1 was ascertained by indirect immunofluorescence assay (IFA). The 5′-flanking region of *Hc-maoc-1* was confirmed to have promoter activity in *C. elegans* and the coding region of *Hc-maoc-1* was also expressed in *C. elegans* to see whether it can influence the growth and development. *Ce-maoc-1* RNAi was performed in *C. elegans* to confirm the function in peroxisomal β-oxidation and accumulation of fat droplets in intestine.

## Methods

### Nematode strains and animals

Diapause, L4, and adults of *H. contortus* (ZJ strain) were collected from sheep abomasa (sheep abomasa were obtained from the Hu Zhou Slaughter house) and stored in liquid nitrogen until use. Adults of *H. contortus* were washed by PBS from the abomasal mucosa. The ingesta, washings and abomsal mucosa were digested in peptic-HCl and then the diapause worms were detected and collected under an anatomical lens (Motic, Fujian, China). L1, L2 and L3 were collected after 1, 3 and 7 days of incubation of collected eggs at 28 °C. Exsheathment of L3 worms (×L3s) were carried out with NaClO as previously described [18]. A *C. elegans* strain of Bristol N_2_ was maintained on Nematode Growth Media (NGM) agar plates at 20 °C [19]. *Caenorhabditis elegans* worms were fed with *Escherichia coli* (OP 50 strain). Worm collections were facilitated with an anatomical lens (Motic, Fujian, China).

### Isolation of *Hc-maoc-1* gene and acquisition 5′-flanking region

The amino acid sequence of *Ce-maoc-1* gene was used to search the Sanger Institute’s *H. contortus* genomic database (http://www.sanger.ac.uk) using BLASTP algorithm. A protein sequence (HCISE00990300.t1_1) with 68% similarity to Ce-MAOC-1 (NP_495494.1) was identified. The coding sequence of *Hc-maoc-1* was amplified using the primer pair *Hc-maoc-1* F and *Hc-maoc-1*R (Additional file 1: Table S1). The PCR reaction procedure was denaturation at 94 °C for 1 min, followed by 35 cycles of 94 °C for 50 s, 52 °C for 40 s, 72 °C for 1 min, with a final extension at 72 °C for 10 min. The purified PCR products were then cloned into pMD18-T vector and sequenced. Gene-specific primers (Additional file 1: Table S1) were designed based on the sequence of 2,000 bp sequences upstream of the *Hc-maoc-1* and used to amplify the upstream region from total genomic DNA of adult *H. contortus.* The PCR reaction procedure was: denaturation at 94 °C for 1 min, followed by 35 cycles of 94 °C for 50 s, 63 °C for 40 s, 72 °C for 2 min, and a final extension at 72 °C for 10 min. The purified PCR products were then cloned into the pMD18-T vector and sequenced.

### Bioinformatic analyses

Homologues of *Hc-maoc-1* gene were identified using the BLASTp at the Nation Center for Biotechnology Information (http://www.ncbi.nlm.nih.gov/Blast). Amino acid sequences were aligned using Clustal W software [20]. Protein motifs were identified by scanning the databases of PROSITE and Pfam (www.ebi.ac.uk/interpro). Phylogenetic analyses were carried out using neighbor-joining (NJ), maximum parsimony (MP) and maximum likelihood (ML) methods, respectively, based on the Jones-Taylor-Thornton (JTT) model in the Molecular Evolutionary Genetic Analysis (MEGA V7) [21].

### Immunofluorescence localisation of Hc-MAOC-1 in the third stage of *H. contortus*

The full-length cDNA of *Hc-maoc-1* was amplified by PCR with primers listed in Additional file 1: Table S1, with restriction sites for endonucleases *Kpn* I and *Hind* III underlined. The PCR products were cloned into pET-30a to construct the prokaryotic expression vector pET-30a*-Hc-maoc-1*, which was then transformed into BL21 (DE3) cells. Recombinant Hc-MAOC-1 (rHc-MAOC-1) expression was induced by 1 mM isopropy β-D-1-thiogalactopyranoside (IPTG) at 37 °C and was purified by affinity chromatography using a Ni-NTA agarose column system (Qiagen, Shanghai, China), according to manufacturer’s protocol. Anti-Hc*-*MAOC-1 polyclonal antibody was produced in a New Zealand white rabbit. Briefly, rHc-MAOC-1 was subcutaneously injected into the rabbit at a concentration of 500 μg/kg every 2 weeks for 3 times. Serum was collected at 7 days after final immunization and antibody titer of each serum was determined by enzyme-linked immunosorbent assay (ELISA).

The indirect immunofluorescence assay (IFA) was performed as described elsewhere [22]. L3 larvae were digested in proteinase K (0.2 mg/ml) at 37 °C for 1 h followed by incubated in 0.1% trition-100 at room temperature overnight. After three washes with 0.01 mM phosphate-buffer saline (PBS), worms were incubated in 1 ml PBS containing 1.5% bovine serum albumin (BSA). After three washes, anti-Hc-MAOC-1 rabbit antibody (dilution 1:1,000) and Alexa Fluor® 488 nm goat anti-rabbit IgG antibody (Invitrogen™) (dilution 1:100) were incubated with worms as primary and secondary antibodies at 37 °C for 1 h, respectively. Worms were then strained with 4′,6-diamidino-2-phenylindole (DAPI) at room temperature for 30 min and viewed by fluorescent microscope (Olympus IX71, Tokyo, Japan).

### Transcriptional analysis of *Hc-maoc-1* in different developmental stages and relative genes in *Ce-maoc-1*RNAi *C. elegans*

Quantitative reverse transcription PCR (qRT-PCR) with specific primers was carried out to determine the relative abundance of *Hc-maoc-1* transcripts in all key stages (i.e. L1s, L2s, iL3s, female L4s, male L4s, female adults and male adults) of *H. contortus* and some transcripts of candidate gene in *Ce-maoc-1*RNAi (RNA interference) *C. elegans*. In brief, total RNA was extracted separately from worms at different developmental stages of *H. contortus* and *Ce-maoc-1*RNAi *C. elegans* employing Trizol reagents (Invitrogen, Shanghai, China), followed by treatment by DNase I (Toyobo, Shanghai, China). First strand cDNA was obtained using ReverTra Ace-α (TOYOBO, Shanghai, China). Gene expression levels were determined by qRT-PCR (25 μl) using SYBR® Green qPCR Master Mix (TOYOBO) and an ABI 7300 thermal cycle. The qRT-PCR reaction procedure was 95 °C for 15 s, 60 °C for 15 s and 72 °C for 30 s for 40 cycles. The dissociation curve was generated under the following conditions: 95 °C for 15 s, 60 °C for 1 min, 95 °C for 15 s and 60 °C for 15 s. Each sample was employed in triplicate using tubulin as an internal loading control using specific primers (Additional file 1: Table S1). The mean threshold cycle (C_q_) values were used for further analysis.

### *Hc-maoc-1* promoter transformation of *C. elegans*

The 5′-flanking region of *Hc-maoc-1* was used as *Hc-maoc-1* promoter and amplified by PCR with primers listed in Additional file 1: Table S1 with restriction sites *Pst* I and *Xba* I underlined. The purified products were cloned into the upstream of the pPD95.77 expression vector’s *gfp* region to verify the ability of initiating the green fluorescent protein (GFP) expression in *C. elegans*. This recombinant plasmid, named as pPD95.77*Hc-maoc-1*-prom, was injected into wild type (N_2_) hermaphrodites using standard gonadal microinjection as described [23]. Another plasmid pRF4 carrying a dominant mutant allele of rol-6 gene was co-injected with the recombinant plasmid each at a final concentration of 50 μg/ml to detect GFP expressed in these transformed *C. elegans*. The parental pPD95.77*Ce-maoc-1-*prom and pRF4 plasmid mixtures were used as controls. The F2 and later generations with a roller phenotype were selectively examined for the expression of GFP by fluorescent microscope (Olympus IX71, Tokyo, Japan).

### Expression of *Hc-maoc-1* in N_2_ strain of *C. elegans*

The full-length cDNA of *Hc-maoc-1* was amplified by PCR with primers containing *Not* I/*Sma* I sites (underlined). The purified PCR products were cloned into pPD95.77 between the 1,574 bp *Ce-maoc-1* promoter region and *gfp* region. The amplification of *Ce-maoc-1* promoter region was performed by PCR from the wild strain genome with the primers containing the restriction site *BamH* I and *Kpn* I (underlined). The recombinant plasmid was designated as CeP-pPD95.77-*Hc-maoc-1* (pPD95.77 vector with *Ce-maoc-1*gene promoter region and whole cDNA region of *Hc-maoc-1* gene). The construct CeP-pPD95.77-*Ce-maoc-1* (pPD95.77 vector with *Ce-maoc-1*gene promoter region and whole cDNA region of *Ce-maoc-1* gene) was used as a control. The full-length cDNA of *Ce-maoc-1* (NP_495494.1) was amplified with primers containing *Not* I/*Sma* I (underlined). The PCR products were cloned into pPD95.77 as described above. The recombinant plasmids were microinjected into N_2_ strain as described above. Transgenic worms with the roller phenotype were selected for examination of GFP activity, lifespan, brood size and body size. Measurement of lifespan, brood size and body size was performed according to Morck, et al. [24, 25]. Body size of worms were gauged from the nose to the tail tip with the free Java image processing program Image J [24]. Larvae was placed singly onto fresh plates and incubated at 20 °C until they had laid the first few eggs. The hermaphrodites were then transferred onto fresh plates daily to prevent overcrowding until egg that laid ceased. Three days after the parents’ removal, the progeny were counted. Five synchronized worms were subsequently plated on these plates for 2 h to lay eggs to measure the lifespan. The adult worms were removed, and then eggs allowed to hatch to larva. Larvae was placed singly onto fresh plates and monitored once daily until death. The animals were transferred once daily while producing eggs to keep them separate from their progeny. Animals were scored as dead when they no longer responded with movement to light prodding of the head. All primers used are listed in Additional file 1: Table S1.

### RNA interference feeding experiments

*Ce-maoc-1* and *Hc-maoc-1* cDNAs were cloned into L4440 vector to generate *Ce-maoc-1*- and *Hc-maoc-1*-specific RNAi vectors. The recombinant plasmids were then transformed into *E. coli* strain HT115 (DE3) cells, an RNase III-deficient *Escherichia coli* strain with isopropyl-β-D-thiogalac-topyranoside-inducible T7 polymerase activity. Primers used for PCR analysis were listed in Additional file 1: Table S1. RNAi plates were prepared according to Kwon and Narasimhan [26]. Young adult *C. elegans* were incubated in the RNAi plates with *E. coli* transformed either with *Ce-maoc-1-* or *Hc-maoc-1-*specific RNAi vector overnight. Parental L4440 vector transformed into the *E. coli* strain HT115 was used as negative controls.

### Lipid staining

Oil-red-O staining was performed as previously described [27]. Briefly, 200–300 *C. elegans* adult worms were collected from the NGM plates and washed with PBS (pH 7.4) three times and settled by gravity. Worms were incubated for 1 h at room temperature with constant rocking in a1:1 ratio of PBS and 2× the Modified Ruvkun’s witches brew (MRWB) buffer with 2% paraformaldehyde (PFA) for permeabilizing the worm cuticle. The 2× MRWB buffer contained 160 mM KCl, 40 mM NaCl, 14 mM Na_2_EGTA, 1 mM spermidine-HCl, 0.4 mM spermine, 30 mM Na-PIPES pH 7.4 and 0.2% β-mercaptoethanol. Worms were then washed with PBS once to remove PFA after being settled by gravity. The worms were subsequently dehydrated in 60% isopropanol for 15 min and strained in 60% Oil-Red-O overnight at room temperature with constant agitation. The stained worms were washed once in PBS containing 0.01% Triton X-100. Afterwards they were mounted on slides and viewed using a microscope with differential interference contrast optics (Nikon, Tokyo, Japan).

### Statistical analyses

Statistical analysis for *Hc-maoc-1* mRNA transcription levels and parameters of *C. elegans* were performed using one-way ANOVA in Excel. *P*-values < 0.05 were considered statistically significant. Graphs were made by GraghPad Prism 5.

## Results

### Characterization of cDNA and phylogenetic analysis of amino acid sequence

**Fig. 1:**
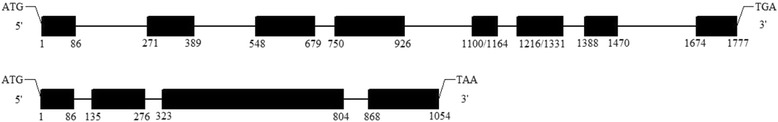
Schematic drawing showing the genomic organization of *maoc-1* of *Haemonchus contortus* (*Hc-maoc-1*) and *C. elegans* (*Ce-maoc-1*). Black boxes represent exons. Lines between the exons represent introns. The number under the black boxes represent the positions in genomes

**Fig. 2:**
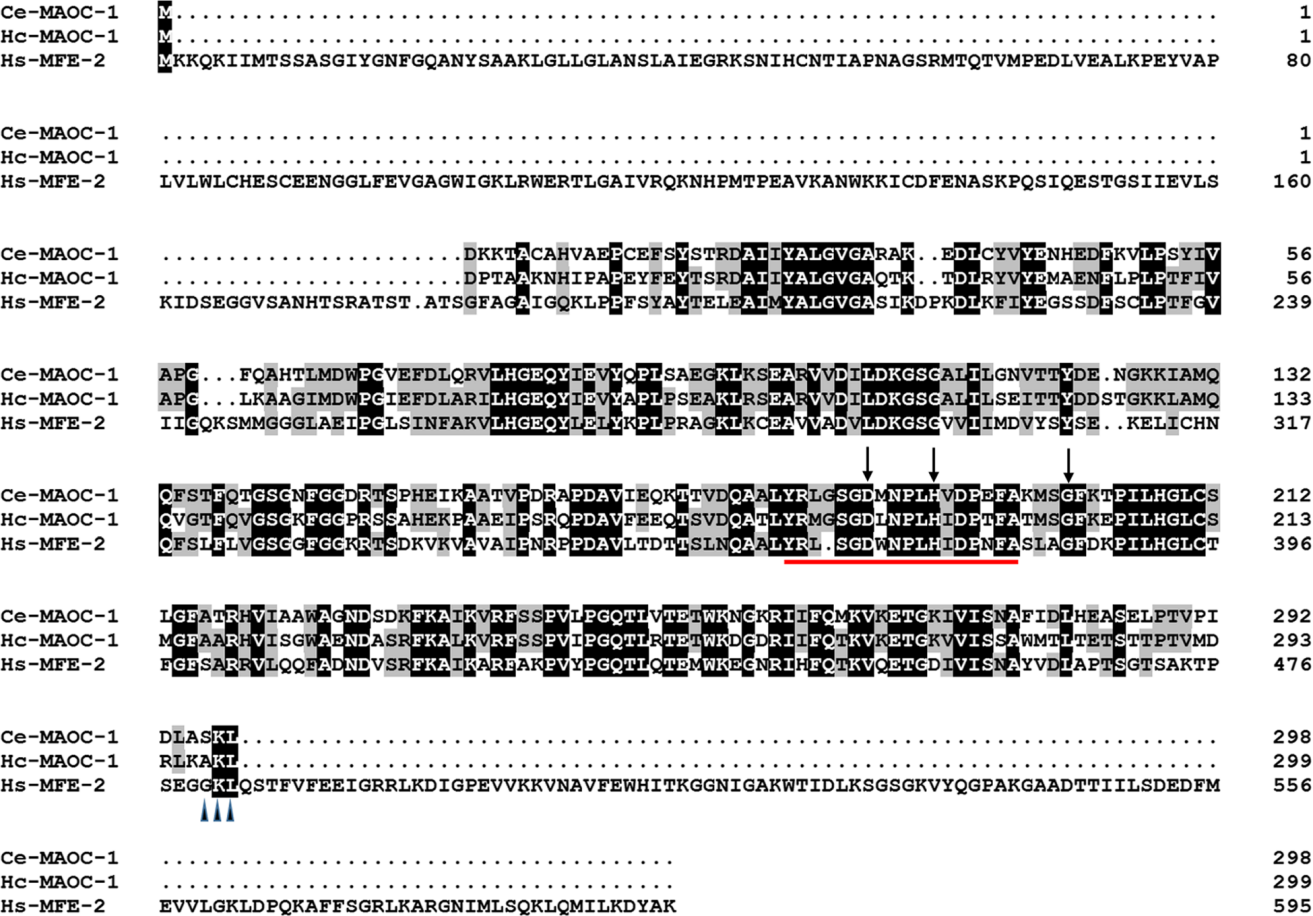
Alignment of the predicted amino acid sequence of *Haemonchus contortus* enyol-CoA hydratase (Hc-MAOC-1) with those of *Caenorhabditis elegans* (Ce-MAOC-1; NP_495494.1) and *Homo sapiens* (Hs-MFE-2;NP_001278957.1). The black line below the sequence alignment indicates the Hydratase 2 motif. Black vertical arrows indicate the conserved catalytic residues of MaoC. Black triangle below the sequence alignment indicates the peroxisomal targeting signal 1

**Fig. 3:**
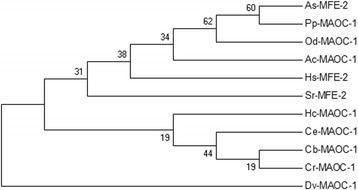
Neighbor-joining tree showing the relationship of *Haemonchus contortus* enyol-CoA hydratase (Hc-MAOC-1) with related protein. The tree was constructed using the Jones-Tayloe-Thornton model in the program MEGA v.7.0 Bootstrap values (> 50%) are shown above or below the branches (1,000 iterations). Shown are the MAOC-1 s of ten organisms including *Ascaris suum* (As), *Pristionchus pacificus* (Pp), *Oesophagostomum dentatum* (Od), *Ancylostoma ceylanicum* (Ac), *Homo sapiens* (Hs), *Strongyloides ratti* (Sr), *Caenorhabditis elegans* (Ce), *Caenorhabditis brenneri* (Cb), *Caenorhabditis remanei* (Cr) and *Dictyocaulus viviparus* (Dv)

The full length of *Hc-maoc-1* (from the ATG initiation codon to the TGA stop codon) was 1,777 bp and contained 8 exons separated by 7 introns (Fig. 1). The gene structure was more complicated compared to *Ce-maoc-1* (Fig. 1). The full-length cDNA of *Hc-maoc-1* was 900 bp in length and encoded a 299-amino-acid protein with a mass of 32.8 KDa, which had 53–84% similarity to homologs from *Caenorhabditis elegans* (NP_495494.1), *Ancylostoma ceylanicum* (EPB80627.1), *Oesophagostomum dentatum* (KHJ91714.1), *Dictyocaulus viviparus* (KJH49452.1) and *Pristionchus pacificus* (KKA69622.1), as well as *Homo sapiens* (NP_001278957.1). The alignment with *C. elegans* and *H. sapiens* showed that Hc-MAOC-1 contained two conservative functional domains, named as the HotDog domain (1–154 bp) and MaoC-like domain (140–282 bp) (Fig. 2). There were several conserved catalytic sites (Asp-187, His-192 and Gly-202) and a conserved hydratase 2 motif (Y-R-L-S-G-D-X-N-XL-H-I-D-P-X-X-A) (Fig. 2). A peroxisome targeting signal 1 (PST1) was observed in the C-domain of Hc-MAOC-1 (Fig. 2). The hypothetical Hc-MAOC-1 protein sequence was phylogenetically analyzed with homologous proteins from 9 nematodes and *Homo sapiens* (Fig. 3). The phylogenetic tree showed that Hc-MAOC-1 was in the same clad with *C. elegans*, which indicated a highly similarity between Hc-MAOC-1 and Ce-MAOC-1 in amino acids.

### Prokaryotic expression of *Hc-maoc-1* and the preparation of its polyclonal antibody

The recombinant plasmid pET-30a*-Hc-maoc-1* highly expressed in *E. coli* BL21 as shown by SDS-PAGE (Additional file 2: Figure S1a). Western blot analysis showed that the purified rHc-MAOC-1 with His tag could be recognized by monoclonal antibody to His tag (Additional file 2: Figure S1b). Western blot was subsequently performed to confirm the polyclonal antibodies could recognize natural Hc-MAOC-1 protein and showed that the natural protein of *H. contortus* reacted with the polyclonal antibodies (Additional file 3: Figure S2).

### Immunofluorescence localisation of Hc-MAOC-1

**Fig. 4:**
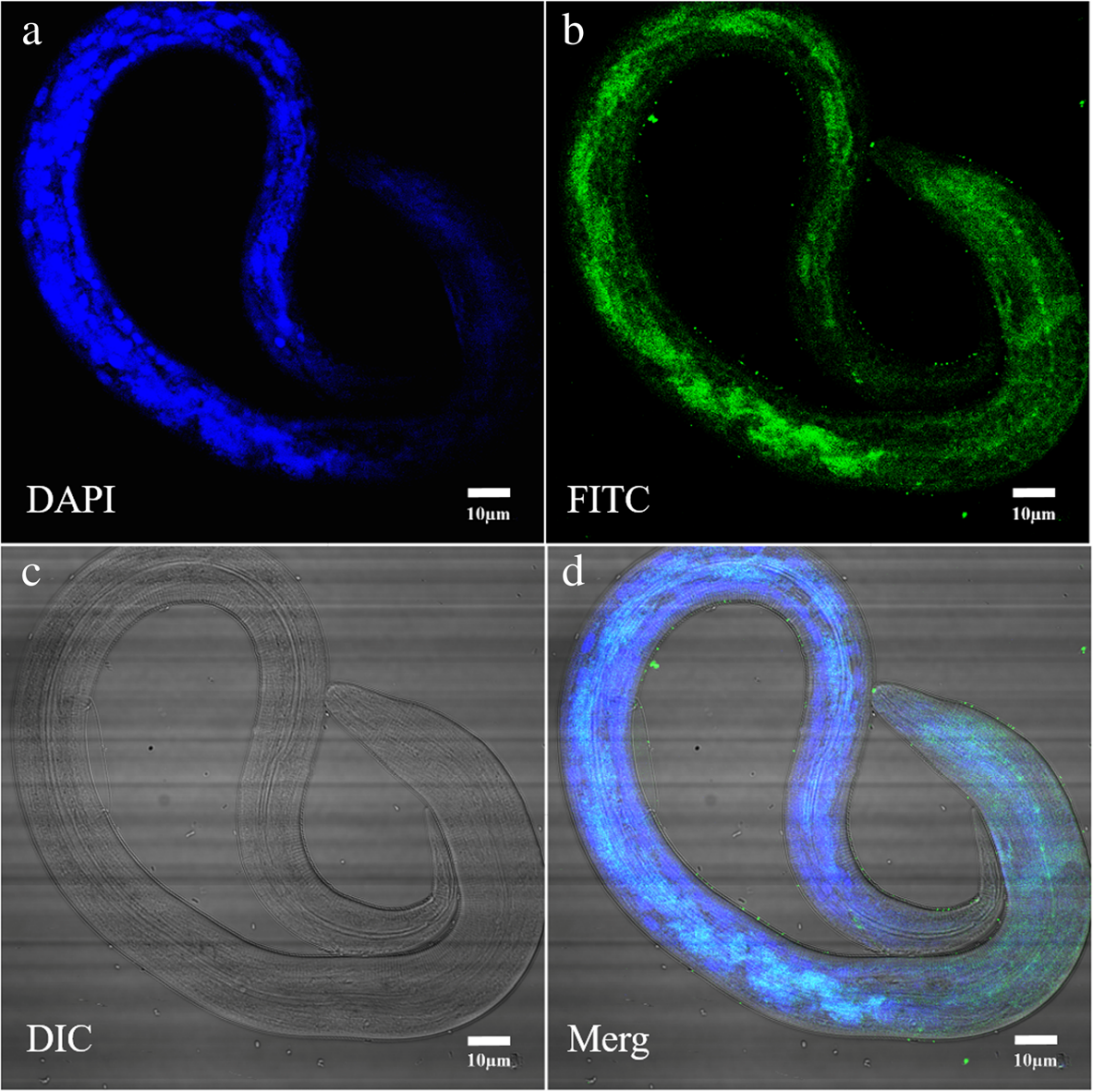
Immunolocalization of Hc-MAOC-1 in the third-stage of *H. contortus*. Panels **a**, **b**, **c** and **d** represent DAPI, FITC, DIC and Merge, respectively. *Scale-bars*: 10 μm

Immunofluorescence localisation revealed that the protein Hc-MAOC-1was present in all cells in *H. contortus* × L3s. The protein Hc-MAOC-1 was detected in the intestinal region and the pharyngeal region as well. Punctiform expression of Hc-MAOC-1 in the intestine region may be the mainly expression pattern of Hc-MAOC-1 in *H. contortus* (Fig. 4)*.*

### Transcriptional level of *Hc-maoc-1* in different developmental stages

**Fig. 5:**
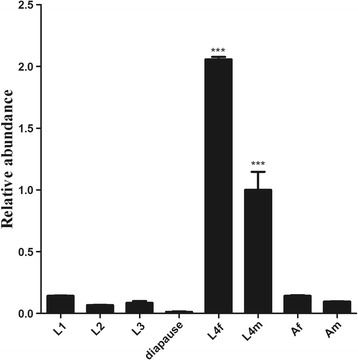
Transcriptional levels of *Hc-maoc-1* at different developmental stages of *H. contortus*. The abundance of *Hc-maoc-1* transcripts was quantified by quantitative real-time PCR (qRT-PCR) in different developmental stages or sexes of *Haemonchus contortus*: first-stage larvae (L1), second-stage larvae (L2), third-stage larvae (L3), diapausing stage (dauer), female fourth-stage (L4f), male fourth-stage (L4m), adult female (Af), and adult male (Am). All gene expression levels were normalized to those of the β-tubulin gene

*Hc-maoc-1* was transcribed at detectable levels at L1, L2, L3, diapause and adults, with a peak in L4 stages. The transcriptional level of *Hc-maoc-1* in diapause stage compared with L1, L2, L3, and adults was significantly downregulated (*t*-test: *t*_(4)_ = 26.79, *P* < 0.0001; *t*_(4)_ = 10.37, *P* = 0.0002; *t*_(4)_ = 4.368, *P* = 0.006; *t*_(4)_ = 18.47, *P* < 0.0001; *t*_(4)_ = 17.82, *P* < 0.0001) (Fig. 5).

### Promoter activity analysis of the 5’-flanking region of *Hc-maoc-1*

**Fig. 6:**
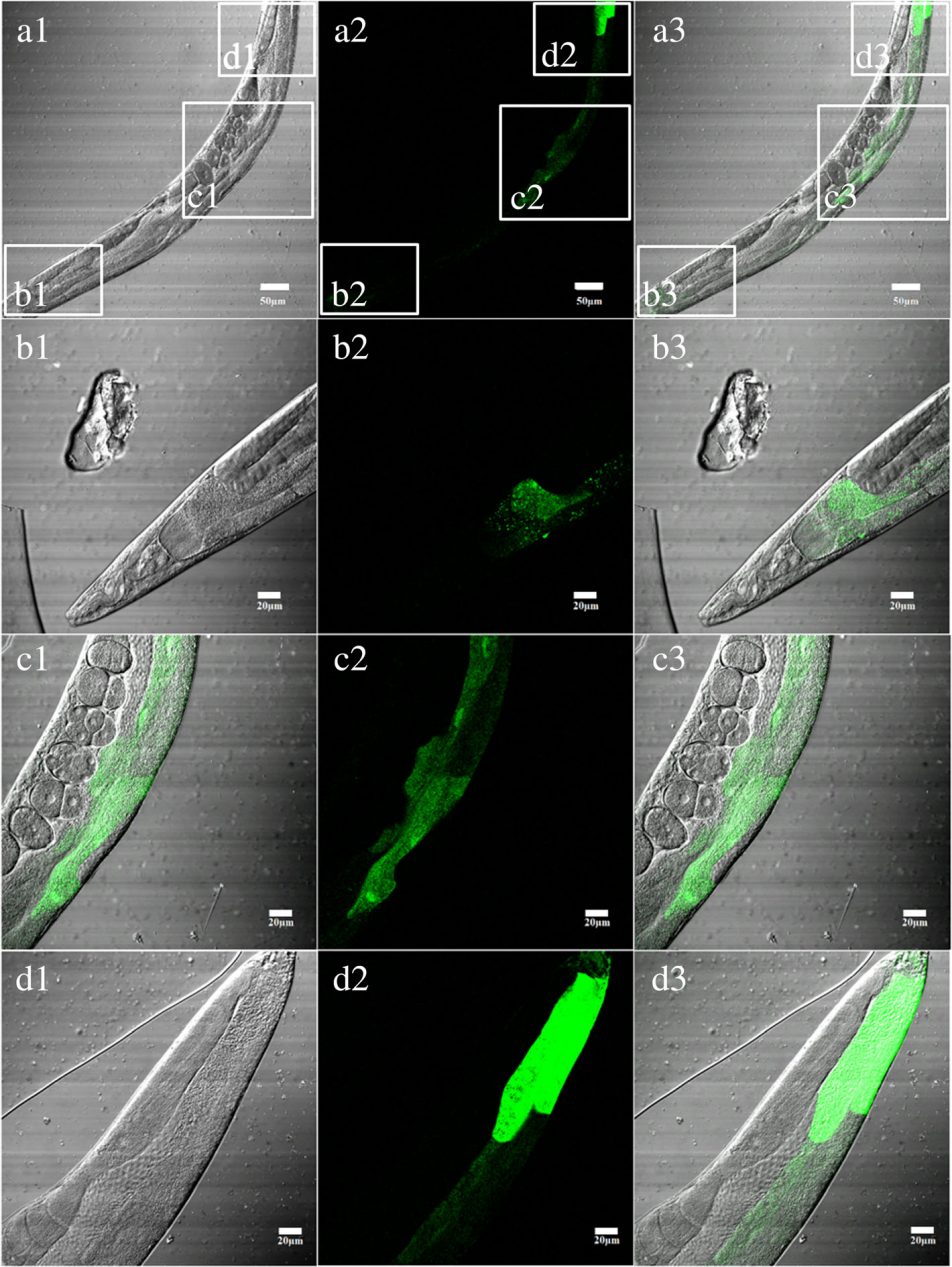
Expression patterns of *Hc-maoc-1* promoter in *C. elegans*. Panel **a** represents transgene worm in 20×. *Scale-bars*: 50 μm. Panels **b**, **c** and **d** represent the different parts of a transgene worm at 40× magnification. DIC, GFP and Merge labelled as 1, 2 and 3, respectively. *Scale-bars*: 20 μm

**Fig. 7:**
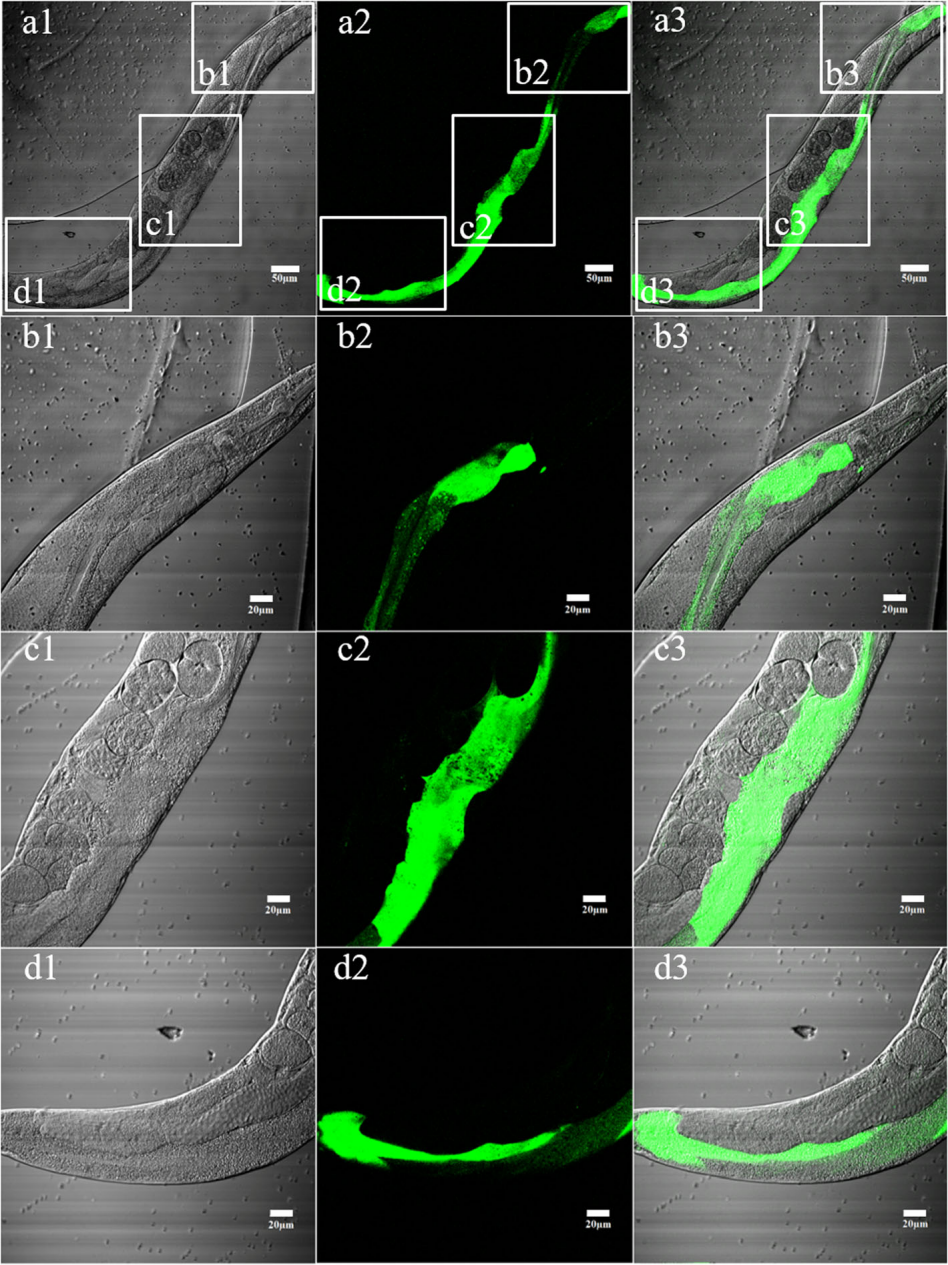
Expression patterns of *Ce-maoc-1* promoter in *C. elegans*. Panel **a** represents transgene worm in 20×. *Scale-bars*: 50 μm. Panels **b**, **c** and **d** represent the different parts of a transgene worm at 40× magnification. DIC, GFP and Merge labelled as 1, 2 and 3, respectively. *Scale-bars*: 20 μm

The reconstructed plasmid described above was transformed into the N2 strain of *C. elegans*. The plasmid pPD95.77*Ce-maoc-1*-prom was microinjected as a control. Transgenic lines showing the roller phenotype were selected. GFP was expressed in the intestine, but the level of GFP expression was obviously enhanced in the distal, middle and anterior part of the intestine region in the transgenic *Hc-maoc-1*-promoter::*gfp* worms (Fig. 6). The GFP expression in the transgenic *Ce-maoc-1-*promoter::*gfp* worms was throughout the intestine, and the level of GFP expression was clearly enhanced in similar part of the intestine. However, compared with the *Hc-maoc-1*-promoter::*gfp* worms, the general expression level of GFP was extremely improved (Fig. 7). The pattern of GFP expression was observed in all developmental stages of *C. elegans* transformed with *Hc-maoc-1-*promoter or *Ce-maoc-1-*promoter (only adults shown).

### Expression of *Hc-maoc-1* in N2 strain of *C. elegans*

**Fig. 8:**
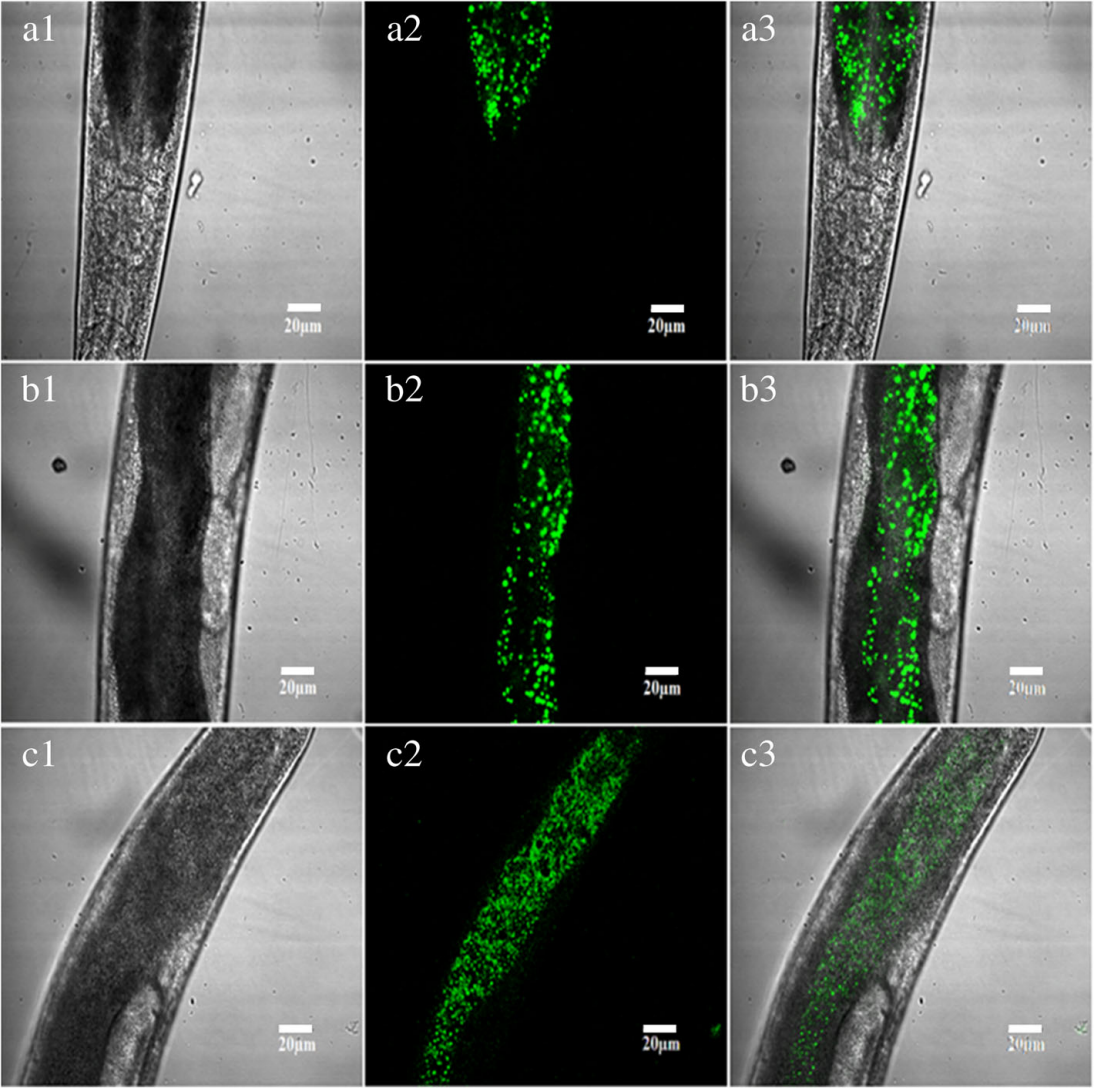
Representative expression patterns of *C. elegans* overexpressing *Hc-maoc-1* was displayed. Panels **a**, **b** and **c** represent the different parts of a transgene worm at 40× magnification. DIC, GFP and Merge labeled as 1, 2 and 3, respectively. *Scale-bars*: 20 μm

**Fig. 9:**
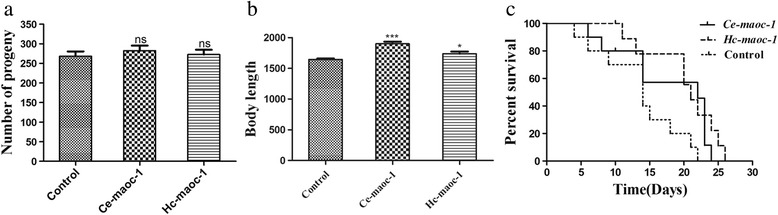
Post-embryonic development and lifespan in *Ce-maoc-1*/*Hc-maoc-1* overexpression *C. elegans* worms. **a** Brood size of 5 worms for three times repeats of overexpressing *Hc-maoc-1* and *Ce-maoc-1*, using pPD95.77 (*Ce-maoc-1-promoter::gfp*) and pRF4 plasmid mixture as a control. **b** Body lengths of 10 worms for three times repeats of *Hc-maoc-1* and *Ce-maoc-1* overexpressed worms. **c** Lifespan of 10 worms for three times repeats of each transgenic line in nematode growth media. Values represent mean ± standard error. *Abbreviation*: ns, not significant. *P < 0.05; ***P < 0.001

The full-length cDNA regions were overexpressed in transgenic *C. elegans* to detect the gene function of *Hc-maoc-1* in vivo. *Ce-maoc-1* 5′-flanking region was used as promoter. The results showed that the expression of *Hc-maoc-1*::*gfp* fusion protein could be observed in the whole intestine with a punctate pattern (Fig. 8). Worms overexpressing *Hc-maoc-1* showed an extended lifespan and significantly lengthened body length (*t*-test: *t*_(18)_ = 2.476, *P* = 0.0234), but there was no significant difference in brood size with N_2_ (*t*-test: *t*_(6)_ = 0.2440, *P* = 0.4077) (Fig. 9).

**Fig. 10:**
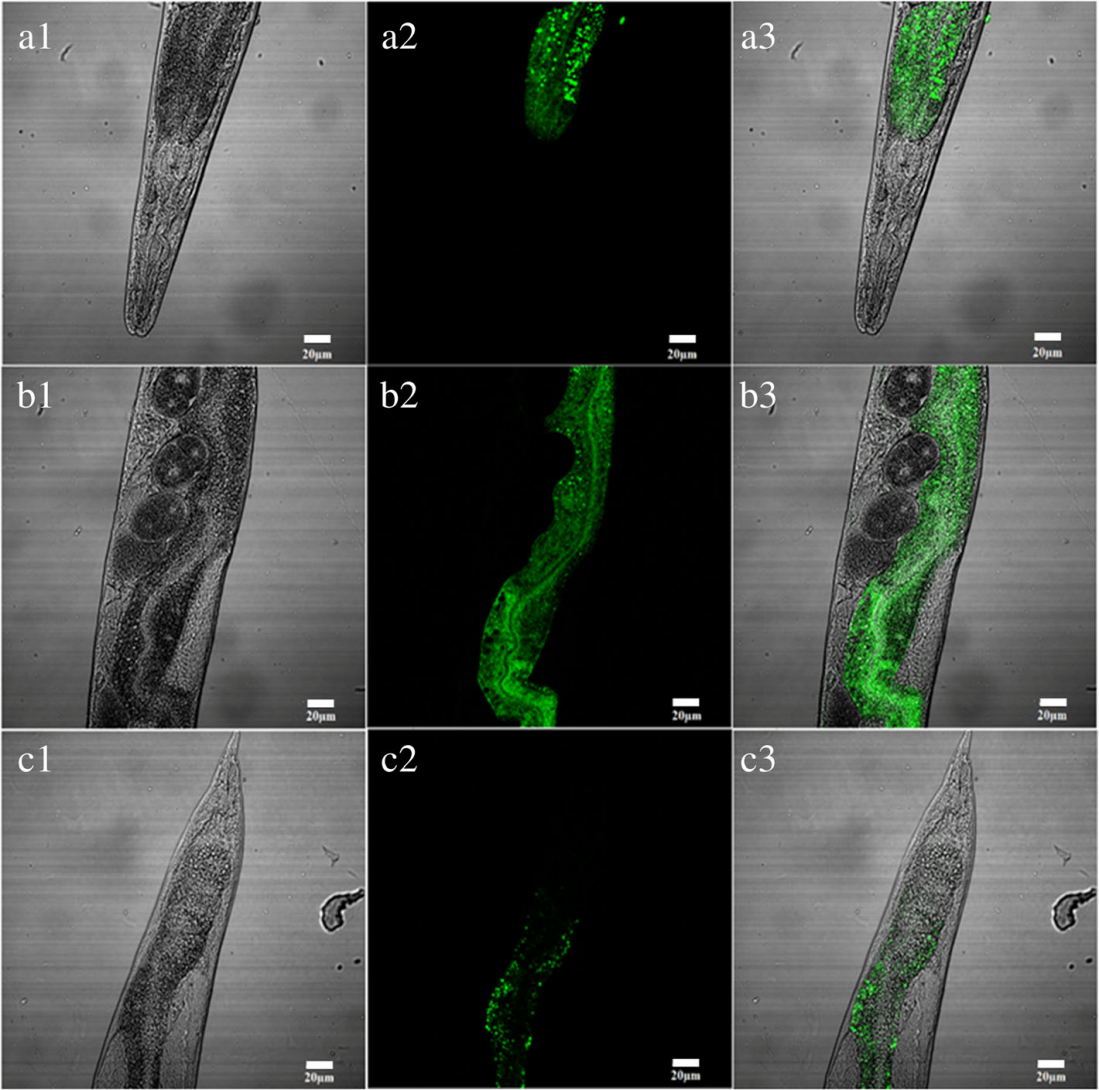
Representative expression patterns of *C. elegans* overexpressing *Ce-maoc-1* was displayed. Panels **a**, **b** and **c** represent the different parts of a transgene worm at 40× magnification. DIC, GFP and Merge labeled as 1, 2 and 3, respectively. *Scale-bars*: 20 μm

The full-length cDNA region of *Ce-maoc-1* was overexpressed as a control. The expression of *Ce-maoc-1*::*gfp* fusion protein in transgenic lines showed the similar results with *Hc-maoc-1*::*gfp* fusion protein (Fig. 10). In addition, there was a significant increase in the body length and lifespan compared to N_2_ and no different in brood size (*t*-test: *t*_(18)_ = 8.685, *P* < 0.0001; *t*_(6)_ = 0.7884, *P* = 0.2329) (Fig. 9).

### RNAi in *C. elegans*

**Fig. 11:**
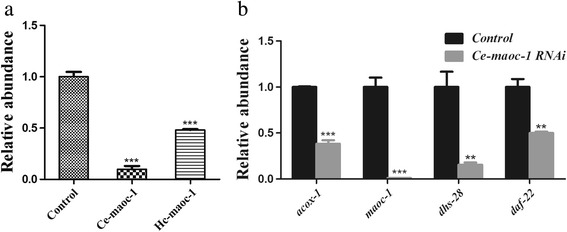
Change in mRNA level of RNA interference with L4440-*Hc-maoc-1* and L4440-*Ce-maoc-1* in N2 *C. elegans*. **a** The abundance of *Ce-maoc-1* transcripts in *Ce-maoc-1*RNAi worms by quantitative real-time PCR (qRT-PCR). ***P < 0.001. **b** The abundance of four genes transcripts in peroxisomal β-oxidation was quantified by quantitative real-time PCR (qRT-PCR) in *Ce-maoc-1* RNAi worms. **P < 0.01; ***P < 0.001

*Hc-maoc-1* transcription was significantly downregulated in diapause-L4s (Fig. 5). To gauge the effect of *Hc-maoc-1* knockdown in *H. contortus*, *Hc-maoc-1*-L4440 and *Ce-maoc-1*-L4440 were constructed and transformed into the HT115 (DE3) cells to perform RNAi in *C. elegans*. qRT-PCR was performed to determine the relative abundance of *maoc-1* transcripts in RNAi worms. The results showed that both *Hc-maoc-1* and *Ce-maoc-1* RNAi could successfully downregulate the *Ce-maoc-1* transcripts in *C. elegans* (*t*-test: *t*_(6)_ = 10.91, *P* < 0.0001; *t*_(6)_ = 15.84, *P* < 0.0001) (Fig. 11). The relevant genes in the peroxisome β-oxidation also showed significant downregulation in the *Ce-maoc-1* RNAi worms (*t*-test: *t*_(4)_ = 15.75, *P* < 0.0001; *t*_(4)_ = 9.646, *P* = 0.0003; *t*_(4)_ = 5.036, *P* = 0.0037; *t*_(4)_ = 5.644, *P* = 0.0024) (Fig. 11).

**Fig. 12:**
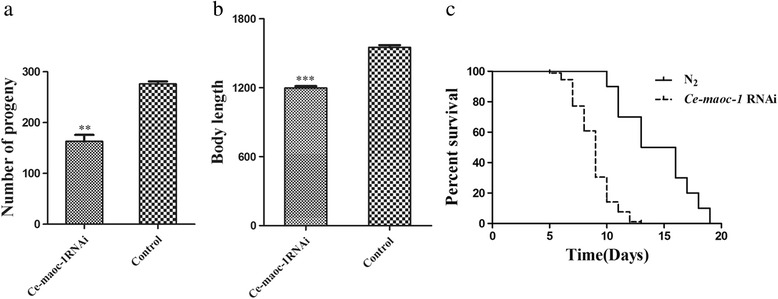
Post-embryonic development and lifespan in *Ce-maoc-1* knockdown *C. elegans* worms. **a** Brood size of 5 worms for three times repeats of *Ce-maoc-1* RNAi worms, using L4440 empty vector-HT115 as a negative control. **b** Body lengths of 10 worms for three times repeats of *Ce-maoc-1* RNAi worms. **c** Lifespan of 10 worms for three times repeats of *Ce-maoc-1* RNAi worms grown on RNAi plates, using L4440 empty vector-HT115 as a negative control. Values represent mean ± standard error. **P < 0.01; ***P < 0.001

**Fig. 13:**
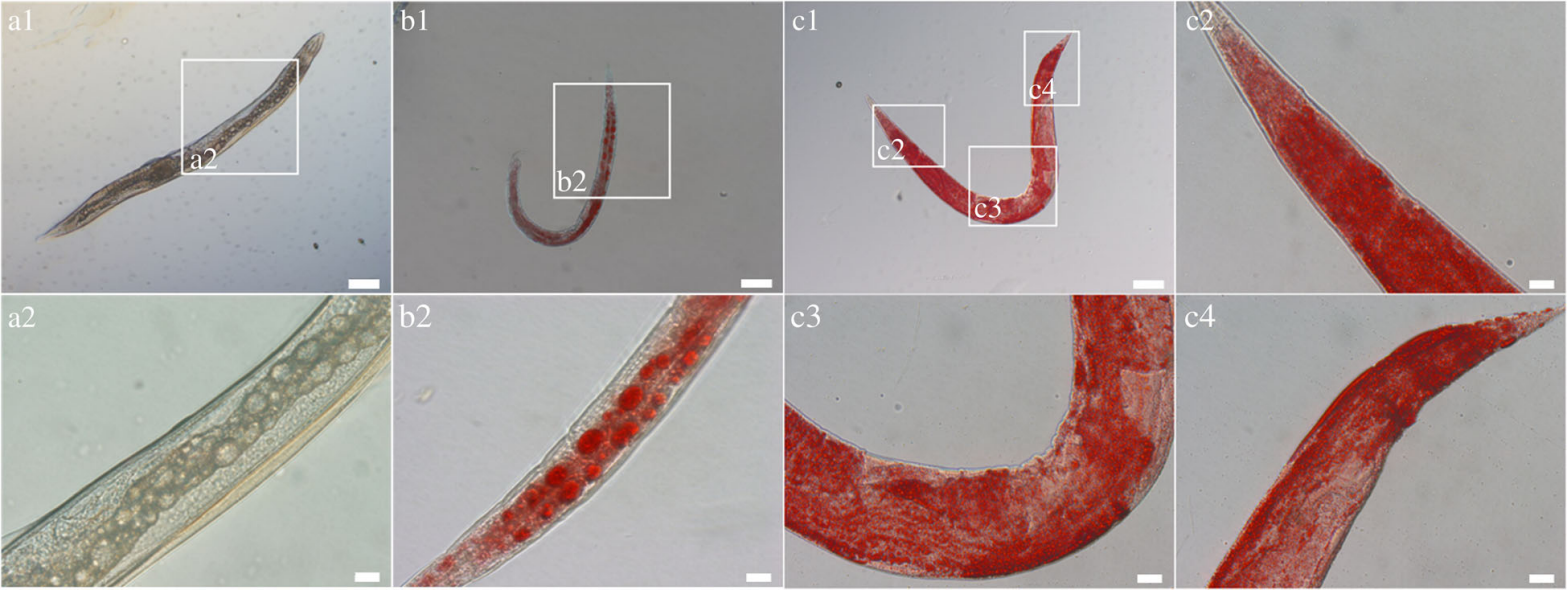
Post-embryonic development of *Ce-maoc-1* RNAi (RNA interference) *C. elegans* worms. **a** DIC of the 7-day-old *Ce-maoc-1*RNAi worm. **b** Oil Red O fat staining of 7-day-old *Ce-maoc-1* RNAi worms. **c** Oil Red O fat staining of 7-day-old N_2_ (L4440 empty vector RNAi widtype *C. elegans* as control). *Scale-bars*: a1, b1, c1, 100 μm; a2, b2, c2, c3, c4, 20 μm

*Ce-maoc-1* RNAi worms showed a significant reduction in the brood size and shortened body length during their life cycle (*t*-test: *t*_(4)_ = 8.184, *P* = 0.0012; *t*_(10)_ = 13.73, *P* < 0.0001). The lifespan of *Ce-maoc-1* RNAi worms were also shortened comparing to the control (Fig. 12). 7 days *Ce-maoc-1* RNAi worms all began to have obvious big fat droplets deposition, which was further confirmed by the Oil-red-O strain (Fig. 13).

## Discussion

*Haemonchus contortus* as a pathogen in ruminants causes great production losses in agricultural industry. Diapausing in the early L4 stage is a strategy for *H. contortus* to survive harsh environments [2]. In *C. elegans*, dauer-inducing pheromone is regarded as the derivatives of the dideoxy-sugar and a signaling molecule inducing L2 larvae to enter the arrested stage [4, 5, 28]. Peroxisome β-oxidation is considered as the pathway to biosynthesize daumones in *C. elegans* [9, 11, 13]. Four peroxisomal enzymes, acyl-CoA oxidase (ACOX) [10], enoyl-CoA hydratase (MAOC-1) [12], (3R)-hydroxyacyl-CoA dehydrogenase (DHS-28) and 3-ketoacyl-CoA thiolase (DAF-22) [9, 11, 29], have been identified to catalyze the four consecutive reactions in the biosynthesis of the menthyl-branched fatty acid moieties of daumones in *C. elegans*. MaoC-like hydratese (MaoC) has been proved to possess the activity of (R)-specific enoyl-CoA hydratase ((R)-hydratese) in associating with the β-oxidation and the polyhydroxyalkanoate (PHA) biosynthetic pathways in the fadB mutant *E. coli* strain [30]. The eukaryotic (R)-hydrateses have similar size, containing about 300 amino acid residues [31].

In this study, we cloned and analysed homologous sequence of *C. elegans*, *Hc-maoc-1.* The full-length cDNA of *Hc-maoc-1* was 900 bp encoding a protein (Hc-MAOC-1) of 299 amino acids, with a calculated molecular mass of 32804.50 Daltons. *Hc-maoc-1* contained eight exons separated by seven introns, which was more complicated than *C. elegans maoc-1*. Sequence and structural analysis showed that Hc-MAOC-1 possesses two conserved domains: HotDog domain and MaoC-like domain (Fig. 2). Hot dog fold, an ancient ubiquitous domain, had been observed in about sixty proteins. The hot dog fold was first described from the β-hydroxydecanoyl thiol eater dehydratase in *E. coli* [32] and the 4-hydroxybenzoyl-CoA thioesterase in *Pseudomaonas sp.* Strain CBS-3 [33]. The motif (Y-R-L-S-G-D-X-N-X-L-H-I-D-P-X-X-A) located in the conserved region was recognized as the hydratase 2 motif first identified on human peroxisomal multifunctional enzyme type 2 [31], and highly conserved in sequence and structure levels [34]. Several residues (Asp-187, His-192 and Gly-202) in the conserved domain had been considered as the important catalytic site in MaoC and its similar eukaryotic hydratases [35]. The peroxisomal targeting signal I (PTS1) in the C-domain of Hc-MAOC-1 and Ce-MAOC-1 was recognized by cytosolic receptors [PEX5 (peroxin)] which escort their cargo protein into the peroxisome [36]. Homologous analysis showed that there was 68% and 53% similarity with homologs from MAOC-1 of *C. elegans* (NP_495494.1) and MFE-2 of *Homo sapiens* (NP_001278957.1), respectively. Additionally, phylogenetic analyses showed that MAOC-1 homologs existed widely in parasitic nematodes, including *A. ceylanicum* (EPB80627.1), *O. dentatum* (KHJ91714.1) and *D. viviparus* (KJH49452.1) (Fig. 3). These findings suggest that MAOC-1 is a relativity conserved protein and might possess similar functions with various parasitic nematodes or free living nematodes.

In the adult nematode, the distribution of peroxisomes was primarily in the epithelial cells of the digestive tract and in the pharyngeal gland [37]. The distribution of Hc*-*MAOC-1 in the structure and tissues of free living stage L3 *H. contortus* was investigated by IFA [22, 38]. As the results show, Hc-MAOC-1 was detected in the intestinal cells with the pattern of punctate fluorescent staining. Additionally, the localization of Hc-MAOC-1 was also presented in all body cells and might also be in the pharyngeal gland (Fig. 4).

The 5′-flanking region of *Hc-maoc-1* was cloned into pPD95.77 to verify its promoter activity and compare it to the 5′-flanking region of *Ce-maoc-1*. The results showed that *Hc-maoc-1-*promoter::*gfp* could expressed throughout the intestine of *C. elegans* (Fig. 6), which indicated that the region possess promoter activity. However, the expression intensity of *Hc-maoc-1-*promoter::*gfp* was weaker than that of *Ce-maoc-1-promoter*::*gfp* in transgenic worms (Fig. 7). Because the promoter region was being forced to function in an alien environment, the relatively GFP levels were lower than control. The micro-injection results also indicated that GFP was localized in the distal, middle and especially anterior part of the intestine. The similar results might be due to possess the same domain: Hotdog and MaoC-like domains and similar gene structure.

Both overexpressing Hc-MAOC-1 and Ce-MAOC-1 showed the punctate expression pattern throughout the intestine. The punctate expression pattern might be a result of the peroxisome targeting signal PTS1 (Fig. 2) [36]. In previous studies, the peroxisome *acox* gene was identified to be expressed mainly in the intestine with a punctate pattern and could be abolished by prx-5 RNAi [10]. Additionally, both *daf-22* and *dhs-28* were expressed mainly in the intestine in *C. elegans* and the intestine was considered as the organ to produce the ascarosides [9]. The results of localization in *H. contortus* and *C. elegans* suggested that the gene *Hc-maoc-1* encode enyol-CoA hydratase in *H. contortus* and might have the same function with *C. elegans*.

qRT-PCR performed to determine the relative abundant of *Hc-maoc-1* transcription in different developmental stages showed that *Hc-maoc-1* transcripts were detected throughout the life-cycle, but had a relative higher abundance in L4 and reduced abundance in diapause stage (Fig. 5). The transcriptional level of *Hc-maoc-1* was detected with a peak in L4 stages. As we know, MAOC-1 is an enzyme in the peroxisome β-oxidation, which participates in the fat acid metabolism and daumone biosynthesis [9]. High transcriptional level of *Hc-maoc-1* in L4 stage might be related to the transition from free living stage to parasitic stage. In the parasitic stage, *H. contortus* began to feed on blood. Since diapause stage was deemed as one form of arrested development [2], the transcriptional level becomes lower than non-diapause stages. The results might hint *Hc-maoc-1* played an important role in the early fourth stage for *H. contortus* to entry into the diapause stages [2]. All these results indicated that Hc-MAOC-1was related to the development of *H. contortus* especially in the L4 and dauer stages.

Peroxisomal metabolism was necessary for postnatal growth, normal maturation and the organisms of embryonic development and organogenesis [39]. Mutations in the human orthologues of the genes in this pathway could cause severe peroxisomal diseases, and patients suffering from this disease showed neurodegeneration and neonatal death [40]. The percentage of adult progeny could be significantly reduced by RNAi inactivation of pex5, pex12, pex13 and pex19 in *C. elegans* [41]. The function of MAOC-1 in peroxisome β-oxidation pathway in very long-chain fatty acid catabolism modulating lipid droplet size was evolutionarily conserved [29]. In this study, there were no significant differences in brood size between *C. elegans* worms overexpressing the *Hc-maoc-1* or *Ce-maoc-1* and N_2_. However, the *Ce-maoc-1* and *Hc-maoc-1* transgenic lines had a lengthened lifespan compared with N_2_ and lengthened body-length. The results might indicate that *Hc-maoc-1* played a more important role in the lifespan and body size, but the role could not be obvious like *Ce-maoc-1*. By contrast, the *Ce-maoc-1*RNAi worms shortened body length and lifespan and had a significant reduction in brood size. These results might indicate specific functions of *Ce-maoc-1* in reproduction, growth and lifespan. Additionally, obvious fat droplet was observed in the gut of *Ce-maoc-1* RNAi worm and identified by the Oil-red-O strain, consistent with Zhang, et al. (Fig. 13) [29]. The significant reduction of relative abundance of four enzymes in peroxisome in the *Ce-maoc-1*RNAi worms suggested the deletion of MAOC-1 in β-oxidation disturb the normal reactions (Fig. 11). The relative quantification of transcriptional levels showed that *Hc-maoc-1* could successfully partially silence the *Ce-maoc-1*. The results may suggest that deletion of *Hc-maoc-1* might also influence reproduction, growth and lifespan.

Our data collectively showed a new gene *Hc-maoc-1*that is a homology to *Ce-maoc-1*. We also ascertained the localization in key developmental stages of *H. contortus* and assessed transcriptional levels of this gene in all stages including dauer stage. Using *C. elegans* as a model to detect the functions of *Hc-maoc-1* showed similarities in functions between *Hc-maoc-1* and *Ce-maoc-1*. Our findings provided new evidence that *H. contortus* might possess the similar molecular mechanism via peroxisome β-oxidation to control dauer phenomenon.

## Conclusions

In this study, a new gene *Hc-maoc-1* was identified which encodes an Enoyl-CoA hydratase in *H. contortus* and was the homologue of *Ce-maoc-1* and human MFE-2. The expression pattern of *Hc-maoc-1* in *H. contortus* was confirmed mainly in the intestine by IFA and a peak transcriptional level in L4 stage. Micro-injection was performed to verify the promoter activity of 5′-flanking region of *Hc-maoc-1* with 5′-flanking region of *Ce-maoc-1* as the control. RNAi inactivation of *Hc-maoc-1* was verified it could partially silence the endogenous *Ce-maoc-1* in N_2_ worms. Measurements with lifespan, brood size and body length showed the similar function to *Ce-maoc-1. Hc-maoc-1* was identified which have the similar characteristics and function, and might play an important role in peroxisomal β-oxidation and development in *H. contortus*.

## Additional file

Additional file 1: Table S1. Primers used in all experiments. (DOCX 15 kb)
Additional file 2: Figure S1. a *Hc-maoc-1*-pET30a transformed Into E. *coli* (BL21) and induced in 37 °C in different temperature. Lane M: Marker; Lane 1: 0 h; Lane 2: 2 h; 3: 4 h; Lane 4: 6 h; Lane 5: 8 h; Lane C: Control (pET30a empty). b Western blot (recombine protein recognized by goat-anti-mouse His Tag antibody). Lane M: Marker; Lane 1: *Hc*-MAOC-1 purified protein with His Tag. (PDF 427 kb)
Additional file 3
**Figure S2**. Western blot: *Hc*-MAOC-1 polyclonal antibody recognized the whole worm protein of *H. contortus. (PDF 374 kb)*

## Abbreviations

ACOX-1: Acyl-CoA oxidase
BSA: Bovine serum albumin
C_q_: Quantification cycle
DAF-22: 3-ketoacyl-CoA thiolase
DAPI: 4′,6′-diamidino-2-phenylindole
DHS-28: (3R)-hydroxyacyl-CoA dehydrogenase
ELISA: Enzyme-linked immunosorbent assay
GFP: Green fluorescent protein
IFA: Indirect immunofluorescence assay
iL3: Infective third-stage larvae
IPTG: Isopropy β-D-1-thiogalactopyranoside
JTT: Jones-Taylor-Thornton
MAOC: Enoyl-CoA hydrate
ML: Maximum likelihood
MP: Maximum parsimony
MRWB: Modified Ruvkun’s witches brew
N_2_: Wild type
NGM: Nematode growth media
NJ: Neighbor-joining
PBS: Phosphate-buffer saline
PCR RNAi: RNA interference
PFA: Paraformaldehyde
PHA: Polyhydroxyalkanoate
PSTI: Peroxisomal targeting signal I
qRT-PCR: Quantitative reverse transcription
rHc-MAOC-1: Recombinant Hc-MAOC-1
xL3s: Exsheathment of L3 worms

## References

[1] Sommerville RI, Davey KG (2002). Diapause in parasitic nematodes*:* a review. Can J Zool.

[2] Blitz NM, Gibbs HC (1971). Morphological characterizatioon of the stage of arrested development of *Haemonchus contortus* in sheep. Can J Zool.

[3] Pelinski M (1980). Seasonal dynamics of stomach nematode invasion in sheep and the effect of inhibition phenomenon of larval development on the sheep infestation in spring. Acta Parasitol Polon.

[4] Golden JW, Riddle DL (1984). The *Caenorhabditis elegans* dauer larva: developmental effects of pheromone, food, and temperature. Dev Biol.

[5] Golden JW, Riddle DL. A pheromone-induced developmental switch in *Caenorhabditis elegans:* Temperature-sensitive mutants reveal a wild-type temperature-dependent process. Proc Natl Acad Sci USA. 1984;81(3):819–23. 10.1073/pnas.81.3.819 PMC344929 6583682

[6] Golden JW, Riddle DL (1982). A pheromone influences larval development in the nematode *Caenorhabditis elegans*. Science.

[7] Butcher RA, Fujita M, Schroeder FC, Clardy J (2007). Small-molecule pheromones that control dauer development in *Caenorhabditis elegans*. Nat Chem Biol.

[8] Jeong PY, Jung M, Yim YH, Kim H, Park M, Hong EM (2005). Chemical structure and biological activity of the *Caenorhabditis elegans* dauer-inducing pheromone. Nature.

[9] Butcher RA, Ragains JR, Li W, Ruvkun G, Clardy J, Mak HY (2009). Biosynthesis of the *Caenorhabditis elegans* dauer pheromone. Proc Natl Acad Sci U S A.

[10] Joo HJ, Kim KY, Yim YH, Jin YX, Kim H, Kim MY (2010). Contribution of the peroxisomal *acox* gene to the dynamic balance of daumone production in *Caenorhabditis elegans*. J Biol Chem.

[11] Joo HJ, Yim YH, Jeong PY, Jin YX, Lee JE, Kim H (2009). *Caenorhabditis elegans* utilizes dauer pheromone biosynthesis to dispose of toxic peroxisomal fatty acids for cellular homoeostasis. Biochem J.

[12] von Reuss SH, Bose N, Srinivasan J, Yim JJ, Judkins JC, Sternberg PW (2012). Comparative metabolomics reveals biogenesis of ascarosides, a modular library of small-molecule signals in *C. elegans*. J Am Chem Soc.

[13] Golden JW, Riddle DL (1985). A gene affecting production of the *Caenorhabditis elegans* dauer-inducing pheromone. Mol Gen Genet.

[14] Srinivasan J, Kaplan F, Ajredini R, Zachariah C, Alborn HT, Teal PE (2008). A blend of small molecules regulates both mating and development in *Caenorhabditis elegans*. Nature.

[15] Zhang X, Feng L, Chinta S, Singh P, Wang Y, Nunnery JK, et al. Acyl-CoA oxidase complexes control the chemical message produced by *Caenorhabditis elegans*. Proc Natl Acad Sci USA. 2015;112(13):3955–60. 10.1073/pnas.1423951112 PMC4386371 25775534

[16] Blaxter M (1998). *Caenorhabditis elegans* is a nematode. Science.

[17] Crook M (2014). The dauer hypothesis and the evolution of parasitism: 20 years on and still going strong. Int J Parasitol.

[18] Rothwell JT, Sangster NC (1993). An in vitro assay utilising parasitic larval *Haemonchus contortus* to detect resistance to closantel and other anthelmintics. Int J Parasitol.

[19] Brenner S (1974). The genetics of *Caenorhabditis elegans*. Genetics.

[20] Thompson JD, Higgins DG, Gibson TJ (1994). CLUSTAL W: improving the sensitivity of progressive multiple sequence alignment through sequence weighting, position-specific gap penalties and weight matrix choice. Nucleic Acids Res.

[21] Kumar S, Stecher G, Tamura K (2016). MEGA7: molecular evolutionary genetics analysis version 7.0 for bigger datasets. Mol Biol Evol.

[22] Riou M, Koch C, Delaleu B, Berthon P, Kerboeuf D (2005). Immunolocalisation of an ABC transporter, P-glycoprotein, in the eggshells and cuticles of free-living and parasitic stages of *Haemonchus contortus*. Parasitol Res.

[23] Mello CC, Kramer JM, Stinchcomb D, Ambros V (1991). Efficient gene transfer in *C. elegans*: extrachromosomal maintenance and integration of transforming sequences. Embo J.

[24] Morck C, Pilon MC (2006). *elegans* feeding defective mutants have shorter body lengths and increased autophagy. Bmc Developmental Biology.

[25] Wong A, Boutis P, Hekimi S (1995). Mutations in the clk-1 gene of *Caenorhabditis elegans* affect developmental and behavioral timing. Genetics.

[26] Kwon ES, Narasimhan D, Yen K, Tissenbaum HA (2010). A new DAF-16 isoform regulates longevity. Nature.

[27] O’Rourke EJ, Soukas AA, Carr CE, Ruvkun G (2009). *C. elegans* major fats are stored in vesicles distinct from lysosome-related organelles. Cell Metab.

[28] Golden JW, Riddle DL (1984). A *Caenorhabditis elegans* dauer-inducing pheromone and an antagonistic component of the food supply. J Chem Ecol.

[29] Zhang SO, Box AC, Xu N, LeMen J, Yu J, Guo F (2010). Genetic and dietary regulation of lipid droplet expansion in *Caenorhabditis elegans*. Proc Natl Acad Sci U S A.

[30] Park SJ, Lee SY (2003). Identification and characterization of a new enoyl coenzyme a hydratase involved in biosynthesis of medium-chain-length polyhydroxyalkanoates in recombinant *Escherichia coli*. J Bacteriol.

[31] Qin YM, Haapalainen AM, Kilpelainen SH, Marttila MS, Koski MK, Glumoff T (2000). Human peroxisomal multifunctional enzyme type 2-Site-directed mutagenesis studies show the importance of two protic residues for 2-enoyl-CoA hydratase 2 activity. J Biol Chem.

[32] Leesong M, Henderson BS, Gillig JR, Schwab JM, Smith JL (1996). Structure of a dehydratase-isomerase from the bacterial pathway for biosynthesis of unsaturated fatty acids: two catalytic activities in one active site. Structure.

[33] Benning MM, Wesenberg G, Liu R, Taylor KL, Dunaway-Mariano D, Holden HM (1998). The three-dimensional structure of 4-hydroxybenzoyl-CoA thioesterase from *Pseudomonas sp.* Strain CBS-3. J Biol Chem.

[34] Pidugu LS, Maity K, Ramaswamy K, Surolia N, Suguna K (2009). Analysis of proteins with the ‘hot dog’ fold: prediction of function and identification of catalytic residues of hypothetical proteins. BMC Struct Biol.

[35] Wang H, Zhang K, Zhu J, Song W, Zhao L, Zhang X (2013). Structure reveals regulatory mechanisms of a MaoC-like hydratase from *Phytophthora capsici* involved in biosynthesis of polyhydroxyalkanoates (PHAs). PLoS One.

[36] Lanyon-Hogg T, Warriner SL, Baker A (2010). Getting a camel through the eye of a needle: the import of folded proteins by peroxisomes. Biol Cell.

[37] Yokota S, Togo SH, Maebuchi M, Bun-Ya M, Haraguchi CM, Kamiryo T (2002). Peroxisomes of the nematode *Caenorhabditis elegans:* distribution and morphological characteristics. Histochem Cell Biol.

[38] De Mendoza MEL, Curtis RHC, Gowen S (1999). Identification and characterization of excreted-secreted products and surface coat antigens of animal and plant-parasitic nematodes. Parasitology.

[39] Van Veldhoven PP, Baes M (2013). Peroxisome deficient invertebrate and vertebrate animal models. Front Physiol.

[40] Steinberg SJ, Dodt G, Raymond GV, Braverman NE, Moser AB, Moser HW (2006). Peroxisome biogenesis disorders. Biochim Biophys Acta.

[41] Petriv OI, Pilgrim DB, Rachubinski RA, Titorenko VI (2002). RNA interference of peroxisome-related genes in *C. elegans:* a new model for human peroxisomal disorders. Physiol Genomics.

