# Supplementary material for: Characterization and function analysis of a novel gene, Hc-maoc-1, in the parasitic nematode Haemonochus contortus
Source: Parasit Vectors. 2017 Feb 6;10:67. doi: 10.1186/s13071-017-1991-1 (PMC5294872; doi:10.1186/s13071-017-1991-1)
Supplement: Additional file 1: Table S1. — Primers used in all experiments. (DOCX 15 kb) [file 13071_2017_1991_MOESM1_ESM.docx]

| **Names** | **Primers 5'–3'** |
| --- | --- |
| *Hc-maoc-1* F | ATG GAT CCT ACA GCA GCT AAG AAT |
| *Hc-maoc-1* R | TCA CAG TTT TGC CTT CAA GCG AT |
| *Hc-maoc-1*PF | ATC AAT CTC TAG CGT GGT CG |
| *Hc-maoc-1*PR | TCT GAA AAA TAG ATG GAG ATA AAC |
| *Hc-maoc-1*Pm F | AACTGCAGATCAATCTCTAGCGTGGTCG |
| *Hc-maoc-1*Pm R | GCTCTAGATTCTGAAAAATAGATGGAGATAAAC |
| *Ce-maoc-1*PmF | CGGGATCCCGTTTCTCTGGAAGAAAAAACC |
| *Ce-maoc-1*PmR | GGGGTACCCCCGGGgcggccgcTTTTAAGTATTTGTCGGAGAAGAAA |
| *Hc-maoc-1*m1F | GGGGTACCATGGATCCTACA GCA GCTAAGAAT |
| *Hc-maoc-1*m1R | CCAAGCTTTTTCACAGTTT TGCCTT CAAGCG AT |
| *Ce-maoc-1*mF | ATTTGCGGCCGC ATG GAT AGA AAA CTG CTT |
| *Ce-maoc-1*mR | TCCCCCGGG TTTTA CAA TTT TGA TGC AAG AT |
| *Hc-maoc-1*Q F | CCT ACAGCAGCTAAGAATCACATC C |
| *Hc-maoc-1*Q R | GTAGACATA TCGGAGATCGGTCTT C |
| tub-F | TGTTCCATCACCCAAGGTATCC |
| tub-R | TGACAGACACAAGGTGGTTGAGAT |
| *Hc-maoc-1*m2F | ATTTGCGGCCGC ATG GAT CCT ACA GCA GCT AAG AAT |
| *Hc-maoc-1*m2R | TCCCCCGGG CAG TTT TGC CTT CAA GCG AT |
| *Ce-maoc-1*F | ATG GAT AGA AAA CTG CTT |
| *Ce-maoc-1*R | TTA CAA TTT TGA TGC AAG AT |
| *Hc-maoc-1*mGF | CCCAAGCTT ATG GAT CCT ACA GCA GCT AAG AAT |
| *Hc-maoc-1*mGR | GGGGTACC TCA CAG TTT TGC CTT CAA GCG AT |
| *Ce-maoc-1*mGF | AACTGCAG ATG GAT AGA AAA CTG CTT |
| *Ce-maoc-1*mGR | AAGCTT TTA CAA TTT TGA TGC AAG AT |
| *Ce-acox-1*QF | GGC AGT GAG AAG ACA GGG GC |
| *Ce-acox-1*QR | TCA GCC ATA AGA GAC ACA TTT |
| *Ce-maoc-1*QF | AGA GCC ATG CGA GTT CTC ATA TTC |
| *Ce-maoc-1*QR | TGA GCTTGAAAT CCT GGA GCA A |
| *Ce-dhs-28*QF | TAT GAC AGC GTT GAG TTT GGA G |
| *Ce-dhs-28*QR | CCG TAC TTC TGA TCT CTC ATG |
| *Ce-daf-22*QF | GTT GGA GTC GGT ATG ACA AAG |
| *Ce-daf-22*QR | CGG TAA GTC CAA CCT CAT ATA G |
| *actin-1*F | GGA ATG TGC AAG GCC GGA T |
| *actin-1*R | ACC TCT CTT GGA TTG GGC CTC |

**Additional file 1: Table S1. Primers used in all experiments**
