# Supplementary material for: Characterization and function analysis of a novel gene, Hc-maoc-1, in the parasitic nematode Haemonochus contortus
Source: Parasit Vectors. 2017 Feb 6;10:67. doi: 10.1186/s13071-017-1991-1 (PMC5294872; doi:10.1186/s13071-017-1991-1)
Supplement: Additional file 2: Figure S1. — a Hc-maoc-1-pET30a transformed Into E. coli (BL21) and induced in 37 °C in different temperature. Lane M: Marker; Lane 1: 0 h; Lane 2: 2 h; 3: 4 h; Lane 4: 6 h; Lane 5: 8 h; Lane C: Control (pET30a empty). b Western blot (recombine protein recognized by goat-anti-mouse His Tag antibody). Lane M: Marker; Lane 1: Hc-MAOC-1 purified protein with His Tag. (PDF 427 kb) [file 13071_2017_1991_MOESM2_ESM.pdf]

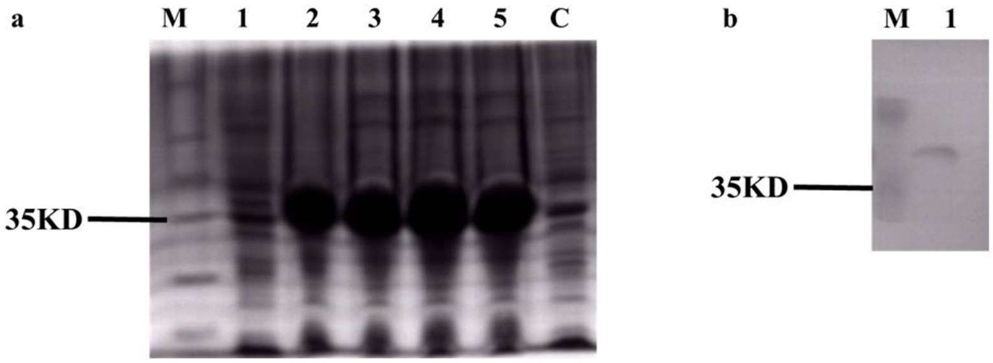

Additional file 2: Figure S1 a *Hc-maoc-1*-pET30a transformed into E. *coli* (BL21) and induced in 37 °C in different temperature. Lane M: Marker; Lane 1: 0 h; Lane 2: 2 h; 3: 4 h; Lane 4: 6 h; Lane 5: 8 h; Lane C: Control (pET30a empty). **b** Western blot (recombine protein recognized by goat-anti-mouse His Tag antibody). Lane M: Marker; Lane 1: *Hc*-MAOC-1 purified protein with His Tag.
