# Supplementary material for: Characterization and function analysis of a novel gene, Hc-maoc-1, in the parasitic nematode Haemonochus contortus
Source: Parasit Vectors. 2017 Feb 6;10:67. doi: 10.1186/s13071-017-1991-1 (PMC5294872; doi:10.1186/s13071-017-1991-1)
Supplement: Additional file 3 — Figure S2. Western blot: Hc-MAOC-1 polyclonal antibody recognized the whole worm protein of H. contortus. (PDF 374 kb) [file 13071_2017_1991_MOESM3_ESM.pdf]

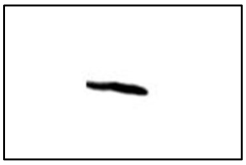

Additional file 3: Figure S2. Western blot**:** *Hc*-MAOC-1 polyclonal antibody recognized the whole worm protein of *H. contortus*
